# Exercise identity and psychological distress among university sport participants: the roles of self-compassion and training stress

**DOI:** 10.3389/fpsyg.2026.1926280

**Published:** 2026-09-21

**Authors:** Runyi Zhang, Haonan Liu, Yang Yu

**Affiliations:** 1 Department of Physical Education, Kyungil University, Gyeongsan-si, Republic of Korea; 2 Department of Physical Education, Yeungnam University College, Daegu, Republic of Korea

**Keywords:** exercise identity, psychological distress, self-compassion, student mental health, training stress, university sport participants

## Abstract

**Introduction:**

University sport participation is often assumed to protect mental health, yet sport can also become an identity-relevant source of pressure. This cross-sectional study examined whether exercise identity was associated with anxiety and depressive symptoms among university sport participants, and whether self-compassion and perceived training stress clarified this association.

**Methods:**

A one-wave online questionnaire yielded 363 eligible participants after consent, eligibility, and attention-check screening.

**Results:**

Exercise identity was positively associated with anxiety and depression, whereas self-compassion was inversely associated with both outcomes and training stress was positively associated with both outcomes. In adjusted complete-case models (*n*= 209), self-compassion showed the most consistent inverse association with symptoms, and this association was stronger under higher perceived training stress. Conditional indirect associations were evident at mean and high training stress. However, sensitivity analyses in the full core-variable sample did not reproduce the exercise identity to self-compassion association, the interaction pattern, or the conditional indirect estimates.

**Discussion:**

These findings challenge a uniformly protective view of sport identity and identify self-compassion and training stress as relevant psychological correlates of distress in university sport contexts. Because the data are cross-sectional and partly complete-case dependent, the conditional indirect model should be interpreted as exploratory.

## Introduction

Sport is often treated as a straightforward mental health resource. This assumption is partly justified: physical activity is associated with lower depressive and anxiety symptoms, intervention evidence in undergraduate samples points in the same direction, public health guidelines now treat regular movement as relevant to physical and mental health, and recent sport psychology reviews link sport participation with wellbeing, positive emotions, and performance (Grasdalsmoen et al., 2020; Huang et al., 2024; Peris-Delcampo et al., 2024; Schuch et al., 2018; World Health Organization, 2020). Organized sport complicates that protective story because it also entails performance standards, repeated evaluation and social comparison, selection uncertainty, intensive training and recovery demands, injury risk, and the need to balance athletic and academic roles. When self-worth, belonging, or future participation depends on performance or physical availability, these features may generate psychological distress even while physical activity remains beneficial. Consensus and meta-analytic evidence therefore show that athlete mental health requires direct assessment rather than optimistic assumptions about sport as protection (Gouttebarge et al., 2019; Reardon et al., 2019). Collegiate athlete studies likewise show that anxiety, depression, and help-seeking difficulties remain common enough to require direct study (Beisecker et al., 2024; Cosh et al., 2024; Edwards et al., 2023a,b; Lee et al., 2021; Rice et al., 2016).

University sport participants sit at this intersection between the health benefits of activity and the demands of organized performance. They train while managing academic pressure, social comparison, evaluation, selection decisions, and periodic injury. Sport is therefore not only a behavior. It is also a valued identity, a social role, and a domain in which young adults learn whether failure is tolerable.

The identity dimension is central because sport-related self-definition can support participation while also increasing vulnerability when the role is threatened. Brewer et al. (1993) framed athletic identity as a role-based self-concept that can shape motivation, social connection, and psychological risk. Exercise identity research extends this logic to mixed samples that include competitive athletes and structured exercisers, making it suitable for university sport participants who may not all hold a formal varsity-team or competitive-athlete position. Recreational participants and structured exercisers can nevertheless identify strongly with the exerciser role (Anderson and Cychosz, 1994; Wilson and Muon, 2008). A strong exercise identity can organize routines, connect students to teammates, and provide a source of competence when sport remains voluntary, socially supportive, and compatible with academic demands.

The same identity can become restrictive when self-worth depends too heavily on training, selection, performance, or bodily readiness. Identity foreclosure is especially relevant during emerging adulthood because students are still developing academic, occupational, and relational identities. A sport identity that supports engagement may therefore coexist with distress when it is paired with pressure, evaluation, injury, or inflexible self-appraisal. This double-edged view leads to a more precise question than whether sport participation is generally beneficial: exercise identity may not show a uniformly protective association with anxiety and depression, and its meaning may depend on how students respond to sport-related difficulty.

Self-compassion is one candidate for clarifying that response. Neff (2003) conceptualized self-compassion through six interconnected components organized as three paired dimensions: self-kindness vs. self-judgment, common humanity vs. isolation, and mindfulness vs. over-identification. In sport, these components may shape appraisal when mistakes, poor performances, injury, and fluctuating motivation invite self-criticism. Self-kindness can replace punitive self-judgment, common humanity can reduce the isolation attached to perceived failure, and mindfulness can help participants notice difficult thoughts without becoming over-identified with them. Together, these processes may support a more specific, changeable appraisal of a setback rather than a global conclusion that the self has failed. Across psychological research, self-compassion is associated with lower anxiety, depression, shame, and maladaptive perfectionism.

Sport-specific evidence supports this placement of self-compassion within the model. A scoping review identified self-compassion as relevant to athletes' responses to setbacks, injury, evaluation, and performance pressure, and studies with women athletes, sport-major students, injured athletes, and caring-climate contexts connect self-compassion with resilience, wellbeing, and adaptive health behavior in sport (Adam et al., 2021; Cormier et al., 2025; Ferguson et al., 2014; Gano-Overway and Peterson, 2023; Mosewich et al., 2011; Wortley and Schellenberg, 2025; Zhang et al., 2023). The Self-Compassion Scale Short Form offers a practical way to assess this construct in a survey with multiple psychological measures (Raes et al., 2011). Theoretically, exercise identity increases the personal relevance of sport outcomes, whereas self-compassion may preserve identity flexibility by separating a difficult performance from global self-worth. Under this appraisal account, the same setback can be treated as specific feedback and a manageable challenge or, when met with self-judgment, isolation, and over-identification, as a broader threat to the self. The present cross-sectional design cannot establish that this process unfolds over time; it can only test whether the proposed associations are consistent with that account.

Training stress provides the boundary condition for this interpretation. Perceived training stress captures subjective strain attached to sport demands, and it is related to but not identical with weekly training hours. Two students may train for the same number of hours while experiencing different levels of pressure, fatigue, coach evaluation, recovery difficulty, or conflict with academic work. Student-athlete stress measurement work separates sport demands from general life stress, and evidence from National Collegiate Athletic Association (NCAA) student-athletes shows that stressors are multi-source rather than limited to training volume (Hwang and Choi, 2016; Lu et al., 2012). Measures such as the College Student-Athletes' Life Stress Scale and athlete recovery-stress instruments make this distinction useful because they assess perceived demands, recovery, and appraisal rather than training load alone (Kellmann and Kallus, 2001; Lu et al., 2012).

Recent studies of athlete stress appraisal show that the way athletes evaluate pressure is associated with both wellbeing and ill-being (Lemelin et al., 2022; Mansell, 2021). Burnout reviews reinforce the same logic: sustained sport demands are associated with motivational, psychological, and physical costs, especially when pressure, recovery problems, and need frustration accumulate (Glandorf et al., 2025; Li et al., 2013; Woods et al., 2025). Training stress may change the strength of the association between self-compassion and distress because high demands reduce recovery, perceived control, and emotional bandwidth while increasing opportunities for harsh self-criticism. The moderation question is therefore not whether stress is beneficial. It is whether self-compassion is more or less strongly associated with symptoms when sport is experienced as demanding.

Current evidence on sport and mental health needs more than another comparison between active and inactive students. Prior work has established that sport and exercise can support wellbeing, but university sport also creates identity-relevant stressors that are not captured by participation status or training volume alone. The present study addresses this gap by examining exercise identity as a sport-related self-concept, self-compassion as a psychological correlate that may clarify how students relate to sport difficulty, and perceived training stress as a boundary condition for the self-compassion to distress association. This framing treats sport participation as a psychological context rather than as a simple protective exposure.

We address one research question and test four hypotheses.

RQ1: What is the association between exercise identity and psychological distress among university sport participants?
H1: Self-compassion is negatively associated with anxiety and depressive symptoms.
H2: Perceived training stress is positively associated with anxiety and depressive symptoms.
H3: Perceived training stress moderates the association between self-compassion and anxiety and depressive symptoms.
H4: The model-based indirect association between exercise identity and psychological distress through self-compassion varies by perceived training stress.

The present cross-sectional study tests this hypothesis-guided but exploratory conditional indirect-association model using observed questionnaire data. Because all variables were measured in one survey wave, the model is used to locate conditional psychological associations rather than to demonstrate temporal process. Figure 1 summarizes the statistical model tested in the study.

**Figure 1:**
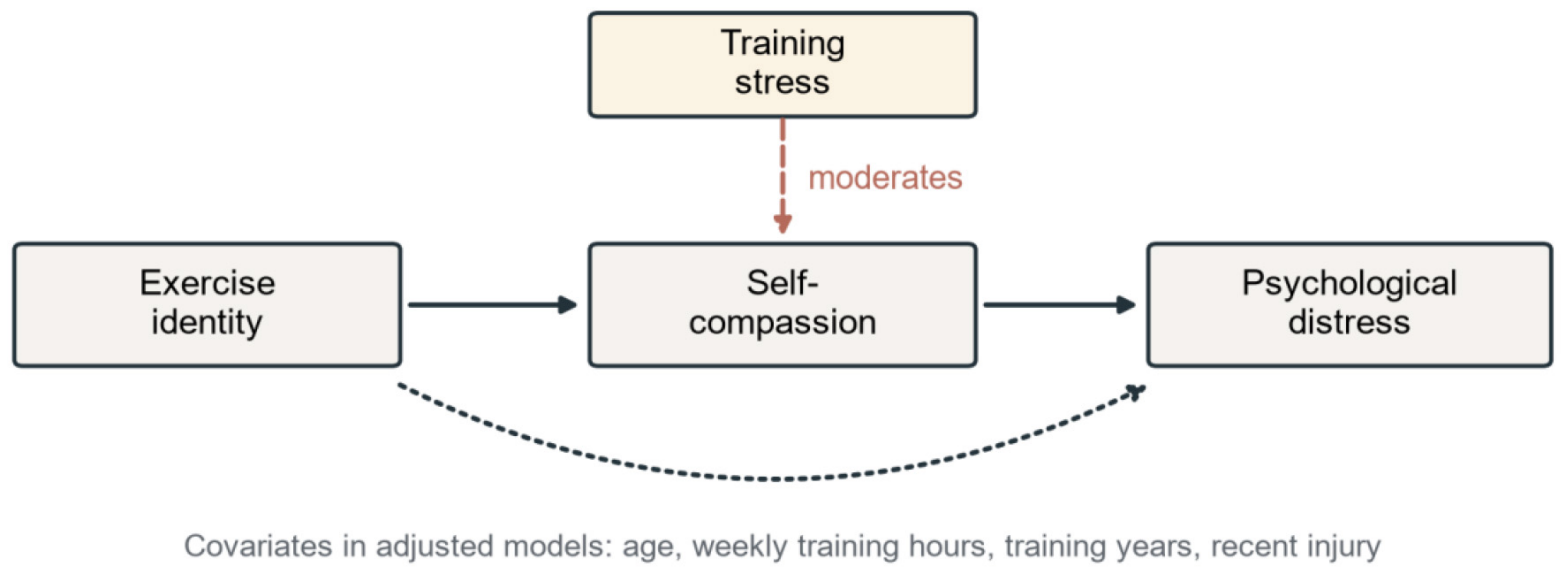
Conceptual model of conditional indirect associations. Exercise identity is modeled as the predictor, self-compassion as the statistical linking variable, anxiety and depressive symptoms as outcomes, and perceived training stress as a moderator of the association between self-compassion and psychological distress. Covariates in the adjusted models include age, weekly training hours, training years, and recent injury status.

## Method

### Design and procedure

This study used a cross-sectional online questionnaire design to examine how exercise identity was associated with psychological distress among university sport participants, and whether self-compassion and perceived training stress clarified that association. The original project plan specified a two-wave survey, but the supplied Excel workbook contained one completed questionnaire wave. The manuscript therefore reports cross-sectional associations and conditional indirect associations, not evidence of temporal ordering or directional change.

Participants completed the questionnaire after indicating informed consent, university-student status, age eligibility, and participation in organized sport or structured exercise. The survey included multi-item self-report measures of exercise identity, self-compassion, perceived training stress, anxiety symptoms, depressive symptoms, and available demographic and sport-participation covariates. The analysis file was de-identified before scoring; platform metadata and IP addresses were not included in the clean dataset used for the manuscript.

### Participants

The Excel workbook contained 619 submitted questionnaires. Participants were retained if they provided consent, reported being university students, reported being at least 18 years old, reported weekly participation in organized sport or structured exercise, and passed the attention-check item. Four hundred cases met the consent and eligibility criteria. Thirty-seven eligible respondents failed the attention check and were excluded. The retained analytic sample included 363 university sport or structured-exercise participants. Because age, weekly training hours, years of sport participation, and recent injury status contained missing values, adjusted regression and conditional indirect-association models used complete cases for all included variables (*n* = 209).

### Measures

Exercise identity was assessed using the nine-item Chinese Exercise Identity Scale reported by Li et al. (2019), which was adapted from the original Exercise Identity Scale developed by Anderson and Cychosz (1994). The items assess the extent to which sport or structured exercise is incorporated into the self-concept. Responses used a 1 to 7 agreement scale, and item scores were averaged so that higher scores indicated stronger exercise identity. The instrument was administered in Chinese, and no new translation or independent validation study was undertaken within the present project; accordingly, internal consistency is reported without treating it as evidence of construct validity. Internal consistency in the retained analytic sample was α = 0.981.

Self-compassion was assessed with 12 author-supplied Chinese short-form items covering self-kindness, common humanity, mindfulness, self-judgment, isolation, and over-identification. Negatively keyed items were reverse scored before averaging, with higher scores indicating greater self-compassion. The short-form structure follows Raes et al. (2011), and exact item wording and adaptation records are available from the authors where scale permissions allow. Internal consistency was α = 0.787.

Perceived training stress was assessed with a five-item study-specific index covering performance pressure, recovery difficulty, academic conflict, emotional strain, and worry about selection, ranking, or evaluation during the past 4 weeks. Responses used a 1–7 agreement scale, and items were averaged. Internal consistency was α = 0.921. Because this measure was constructed for the present project, it is described as a study-specific index rather than a previously validated scale.

Anxiety symptoms were assessed with seven past-2-week symptom items using a 0 to 3 frequency scale consistent with the GAD-7 symptom format (Spitzer et al., 2006). Depressive symptoms were assessed with eight past-2-week symptom items using the same response scale consistent with the PHQ-8 format (Kroenke et al., 2009). Symptom items were summed, with higher scores indicating greater psychological distress. Internal consistency was α = 0.975 for anxiety and α = 0.968 for depression. Measurement and scoring details are summarized in Table S1.

Covariates available in the export included age, gender, sport level, sport type, weekly training hours, years of sport participation, competition status, and recent injury. The adjusted primary models included age, weekly training hours, years of sport participation, and recent injury status because these variables directly indexed training exposure or health disruption and were available in analyzable form. Categorical sport-participation descriptors were summarized to characterize the sample.

### Data analysis

The Excel workbook was converted into de-identified files in comma-separated values (CSV) format using the project data-processing script. Text response options were recoded into numeric item scores, negatively keyed self-compassion items were reverse scored, and construct scores were computed using the planned scoring rules. Descriptive statistics, reliability coefficients, and Pearson correlations were calculated for the retained analytic sample. The study analyzed an existing one-wave dataset; no *a priori* sample-size or power analysis was conducted for the present moderation and conditional indirect-association models. We therefore emphasize effect estimates, uncertainty intervals, and the loss of observations in complete-case models rather than using retrospective observed-power calculations.

The primary model specified exercise identity as the focal predictor, self-compassion as the statistical linking variable, perceived training stress as the moderator of the self-compassion to symptom association, and anxiety and depression as indicators of psychological distress. Continuous predictors were mean-centered before estimating the adjusted regression models. The self-compassion model predicted self-compassion from exercise identity and covariates. Each outcome model predicted anxiety or depression from exercise identity, self-compassion, perceived training stress, the self-compassion by training-stress interaction, and covariates. H1 and H2 were evaluated from the directions and two-sided significance tests of the corresponding Pearson correlations. H3 was evaluated by the self-compassion by training-stress interaction coefficient in each adjusted outcome model. Conditional indirect associations for H4 were computed as the exercise identity to self-compassion coefficient multiplied by the conditional self-compassion to distress association at sample-derived low (−1 SD), mean, and high (+1 SD) training stress. In the adjusted complete-case sample, these values were 3.47, 4.72, and 5.98 on the 1–7 scale; they are descriptive probe values, not validated clinical or categorical cutoffs. Percentile bootstrap 95% confidence intervals used 2,000 resamples where model estimation succeeded, and an indirect estimate was treated as statistically distinguishable from zero when its interval excluded zero. Regression results are reported as unstandardized coefficients, standard errors, *t* statistics, and two-sided *p* values using α = 0.05. Because the hypotheses were specified in advance and the two symptom outcomes were highly correlated, no multiplicity adjustment was applied; results are interpreted by their overall pattern and sensitivity analyses rather than isolated *p* values.

No imputation was used for the adjusted primary models. Because covariate availability reduced the adjusted model sample to 209 complete cases, a sensitivity analysis repeated the core-variable models in the retained sample without the age, training-hours, training-years, and injury covariates. Complete-case selection was evaluated descriptively by comparing participants retained in the adjusted models with retained analytic-sample participants who lacked at least one adjusted-model covariate. This sensitivity comparison changed both sample composition (*n* = 209 vs. *n* = 363) and covariate specification; it therefore assesses joint robustness but cannot identify whether discrepant estimates arise from complete-case selection, covariate adjustment, or both. Because all variables were measured in one survey wave, the conditional indirect estimates are interpreted as model-based associations rather than evidence that exercise identity changes self-compassion over time or that self-compassion later changes symptoms.

## Results

### Data screening and descriptive statistics

The Excel file contained 619 submitted questionnaires. Four hundred cases met the consent, age, student-status, and sport-participation screening criteria. After excluding 37 eligible respondents who failed the attention-check item, 363 cases were retained for analysis. Figure S1 summarizes the sample flow. The available file was a one-wave export, so the analyses below treat all variables as cross-sectional self-report measures rather than as a two-wave design.

Participants had a mean age of 21.65 years (SD = 3.59; *n* = 325). They reported 6.37 weekly training hours on average (SD = 4.70; *n* = 219) and 5.66 years of regular sport or exercise participation (SD = 5.00; *n* = 276). The retained sample was 52.3% female; 59.2% reported recreational participation and 19.3% reported university-team participation. Team sports (36.1%) and individual sports (33.1%) were the two largest sport-type groups, 28.4% reported formal competition in the past semester, and 31.4% reported a recent injury. Sample characteristics are reported in Table 1, with categorical response counts provided in Table S2. The adjusted primary models used 209 complete cases because some covariates contained missing values.

**Table 1:** Sample characteristics and sport-participation profile.

Characteristic	Value
Submitted responses	619
Eligible and consenting before attention-check exclusion	400
Retained analytic sample	363
Age, years	21.65 (3.59); *n* = 325
Gender, female	190 (52.3%)
Weekly training hours	6.37 (4.70); *n* = 219
Training experience, years	5.66 (5.00); *n* = 276
Sport level, recreational participation	215 (59.2%)
Sport level, university team	70 (19.3%)
Sport type, team sport	131 (36.1%)
Sport type, individual sport	120 (33.1%)
Formal competition in past semester	103 (28.4%)
Recent injury	114 (31.4%)
Complete cases for adjusted primary models	209

Values are *n, n* (%), or *M* (SD). Categorical labels are translated from the questionnaire response options. The adjusted primary models use complete cases for age, weekly training hours, training years, recent injury, and the five core study variables.

Complete-case comparisons indicated that the adjusted-model subsample was not exchangeable with the retained participants excluded for covariate missingness. Complete cases reported higher exercise identity (*M* = 5.93 vs. 5.11), perceived training stress (*M* = 4.72 vs. 4.12), anxiety symptoms (*M* = 9.68 vs. 5.70), and depressive symptoms (*M* = 9.30 vs. 5.04), whereas self-compassion did not differ materially (*M* = 3.41 vs. 3.37). These comparisons are reported in Table S4 and are treated as evidence that the adjusted complete-case models describe a higher-strain subset of the retained sample.

The five core study variables covered broad portions of their observed score ranges. Mean exercise identity was high (*M* = 5.59, SD = 1.16 on a 1–7 scale), whereas mean self-compassion was moderate (*M* = 3.40, SD = 0.52 on a 1–5 scale). Participants reported moderate perceived training stress (*M* = 4.47, SD = 1.09 on a 1–7 scale), with mean anxiety and depressive symptom scores of 7.99 (SD = 6.03) and 7.49 (SD = 6.23), respectively. Figure 2 displays the distributions of the five core study variables. These descriptive distributions are not presented as a substitute for an *a priori* power analysis or as evidence that every modeled effect was estimated with adequate precision.

**Figure 2:**
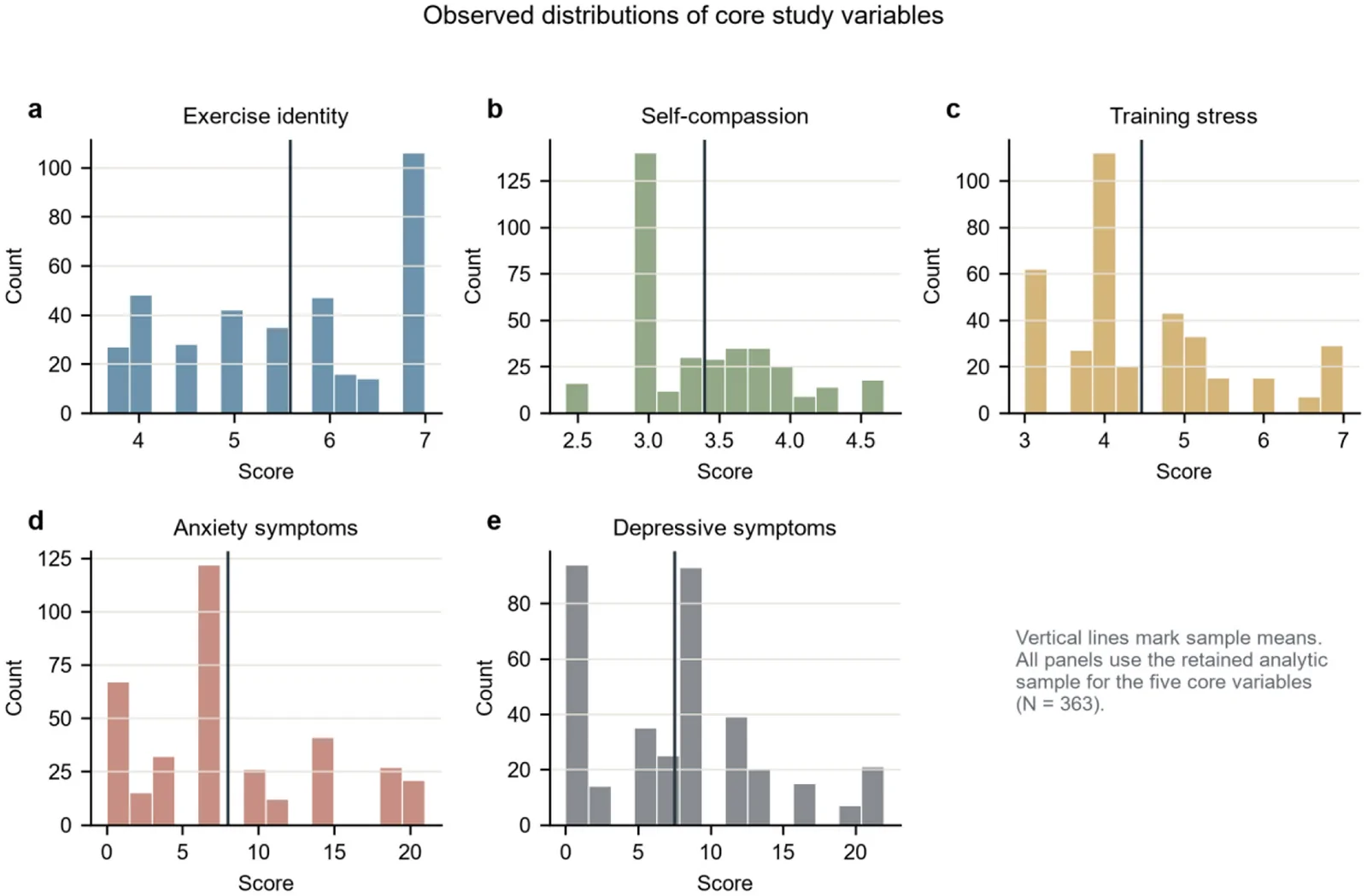
Observed distributions of core study variables. **(a)** Exercise identity, **(b)** self-compassion, **(c)** perceived training stress, **(d)** anxiety symptoms, and **(e)** depressive symptoms. The histograms show the retained analytic sample (*N* = 363), and the vertical lines indicate sample means.

### Reliability and correlations among study variables

Internal consistency was acceptable for exercise identity (α = 0.981), self-compassion (α = 0.787), training stress (α = 0.921), anxiety (α = 0.975), and depression (α = 0.968). Exercise identity was not correlated with self-compassion, *r* = 0.029, *p* = 0.579, but it was positively correlated with perceived training stress, *r* = 0.277, *p* < 0.001, anxiety, *r* = 0.348, *p* < 0.001, and depression, *r* = 0.408, *p* < 0.001. For RQ1, the bivariate evidence therefore indicated a positive exercise identity-distress association rather than a protective association. Self-compassion correlated negatively with anxiety, *r* = −0.506, *p* < 0.001, and depression, *r* = −0.463, *p* < 0.001, supporting H1 at the correlational level. Perceived training stress correlated positively with anxiety, *r* = 0.610, *p* < 0.001, and depression, *r* = 0.499, *p* < 0.001, supporting H2 at the correlational level. Anxiety and depressive symptoms were strongly correlated, *r* = 0.938, *p* < 0.001, indicating that the two outcomes were closely related indicators of internalizing distress. Descriptive statistics, reliability estimates, and correlations are reported in Tables 2 and 3; the same correlation pattern is visualized in Figure S2.

**Table 2:** Descriptive statistics and reliability.

Variable	*n*	*M*	SD	Min	Max	α
Exercise identity	363	5.59	1.16	3.67	7.00	0.981
Self-compassion	363	3.40	0.52	2.42	4.67	0.787
Perceived training stress	363	4.47	1.09	3.00	7.00	0.921
Anxiety symptoms	363	7.99	6.03	0.00	21.00	0.975
Depressive symptoms	363	7.49	6.23	0.00	22.00	0.968

Internal consistency is Cronbach's α.

**Table 3:** Pearson correlations among study variables.

Variable	Exercise identity	Self-compassion	Training stress	Anxiety	Depression
Exercise identity	—	—	—	—	—
Self-compassion	0.029	—	—	—	—
Training stress	0.277^***^	−0.183^***^	—	—	—
Anxiety	0.348^***^	−0.506^***^	0.610^***^	—	—
Depression	0.408^***^	−0.463^***^	0.499^***^	0.938^***^	—

^***^p < 0.001.

### Conditional indirect-association models

The adjusted self-compassion model explained 67.4% of the variance in self-compassion. In this model, exercise identity was associated with self-compassion, *b* = 0.095, SE = 0.020, *p* < 0.001, after adjustment for age, weekly training hours, training years, and recent injury. This adjusted association should be read alongside the near-zero bivariate correlation between exercise identity and self-compassion, because the positive coefficient emerged only after accounting for covariates.

The adjusted anxiety model explained 75.3% of the variance in anxiety symptoms. Exercise identity was not directly associated with anxiety after covariate adjustment, *b* = 0.054, SE = 0.311, *p* = 0.861. Self-compassion was associated with lower anxiety, *b* = −9.970, SE = 0.777, *p* < 0.001, and the self-compassion by training-stress interaction was significant, *b* = −8.734, SE = 1.282, *p* < 0.001. Perceived training stress showed a positive but non-significant conditional coefficient at the centered value of self-compassion, *b* = 0.577, SE = 0.319, *p* = 0.072.

In the depression model, which explained 76.7% of the variance in depressive symptoms, exercise identity remained positively associated with depression after adjustment, *b* = 0.769, SE = 0.303, *p* = 0.012. Self-compassion was associated with lower depression, *b* = −13.098, SE = 0.758, *p* < 0.001, and the self-compassion by training-stress interaction was significant, *b* = −8.840, SE = 1.251, *p* < 0.001. The conditional training-stress coefficient in this centered interaction model was negative, *b* = −0.922, SE = 0.311, *p* = 0.003; this coefficient should not be interpreted as evidence that training stress is generally protective, because the bivariate association between training stress and depression was positive and the regression coefficient is conditional on centered self-compassion, the interaction term, and covariates. Adjusted model estimates are reported in Table 4, and the model-implied self-compassion by training-stress interaction is shown in Figure S3. In the adjusted complete-case models, Table 4 and Figure S3 therefore supported H3 for both anxiety and depression.

**Table 4:** Conditional indirect-association model estimates.

Model	Term	*b*	SE	*t*	*p*
Self-compassion	Intercept	3.412	0.02	171.087	< 0.001
Self-compassion	Exercise identity	0.095	0.02	4.724	< 0.001
Self-compassion	Age	0.12	0.007	17.659	< 0.001
Self-compassion	Weekly training hours	−0.045	0.005	−9.413	< 0.001
Self-compassion	Training years	−0.017	0.004	−4.133	< 0.001
Self-compassion	Recent injury	0.325	0.043	7.534	< 0.001
Anxiety	Intercept	9.014	0.232	38.906	< 0.001
Anxiety	Exercise identity	0.054	0.311	0.175	0.861
Anxiety	Self-compassion	−9.97	0.777	−12.828	< 0.001
Anxiety	Training stress	0.577	0.319	1.81	0.072
Anxiety	Self-compassion × training stress	−8.734	1.282	−6.812	< 0.001
Anxiety	Age	1.264	0.13	9.746	< 0.001
Anxiety	Weekly training hours	0.071	0.062	1.134	0.258
Anxiety	Training years	−0.183	0.054	−3.393	0.001
Anxiety	Recent injury	1.253	0.566	2.216	0.028
Depression	Intercept	8.628	0.226	38.159	< 0.001
Depression	Exercise identity	0.769	0.303	2.533	0.012
Depression	Self-compassion	−13.098	0.758	−17.268	< 0.001
Depression	Training stress	−0.922	0.311	−2.965	0.003
Depression	Self-compassion × training stress	−8.84	1.251	−7.065	< 0.001
Depression	Age	1.944	0.127	15.359	< 0.001
Depression	Weekly training hours	0.062	0.061	1.015	0.311
Depression	Training years	−0.237	0.053	−4.518	< 0.001
Depression	Recent injury	3.773	0.552	6.835	< 0.001

Continuous predictors were mean-centered. Adjusted-model complete-case *n* = 209. Interaction terms provide the formal tests of H3; two-sided α = 0.05.

Bootstrap conditional indirect estimates varied across sample-derived levels of training stress: low (−1 SD = 3.47), mean (4.72), and high (+1 SD = 5.98) in the adjusted complete-case sample. These values are relative to this sample and are not established cutoffs for low, moderate, or high training stress. For anxiety, the indirect estimate was 0.092 at low stress, −0.943 at mean stress, and −1.978 at high stress. The low-stress confidence interval crossed zero, whereas the mean- and high-stress confidence intervals did not. For depression, the corresponding estimates were −0.192, −1.239, and −2.286, again with confidence intervals excluding zero at mean and high training stress but not at low training stress. Confidence intervals are reported in Table 5, and Figure 3 displays the conditional association pattern. These adjusted complete-case estimates supported H4 only at mean and high training stress. Because the design is cross-sectional, these estimates should be interpreted as conditional indirect associations rather than evidence of temporal process.

**Table 5:** Conditional indirect associations at different levels of training stress.

Outcome	Training stress	Indirect effect	95% CI low	95% CI high	Bootstrap draws
Anxiety	Low (−1 SD; 3.47)	0.092	−0.297	0.564	2,000
Anxiety	Mean (4.72)	−0.943	−1.212	−0.638	2,000
Anxiety	High (+1 SD; 5.98)	−1.978	−2.642	−1.306	2,000
Depression	Low (−1 SD; 3.47)	−0.192	−0.667	0.299	2,000
Depression	Mean (4.72)	−1.239	−1.576	−0.834	2,000
Depression	High (+1 SD; 5.98)	−2.286	−3.102	−1.493	2,000

Bootstrap percentile 95% CIs used 2,000 resamples where model estimation succeeded. Low, mean, and high training stress are sample-derived probe values (3.47, 4.72, and 5.98), not validated cutoffs. Estimates are conditional indirect associations, not causal mediation effects.

**Figure 3:**
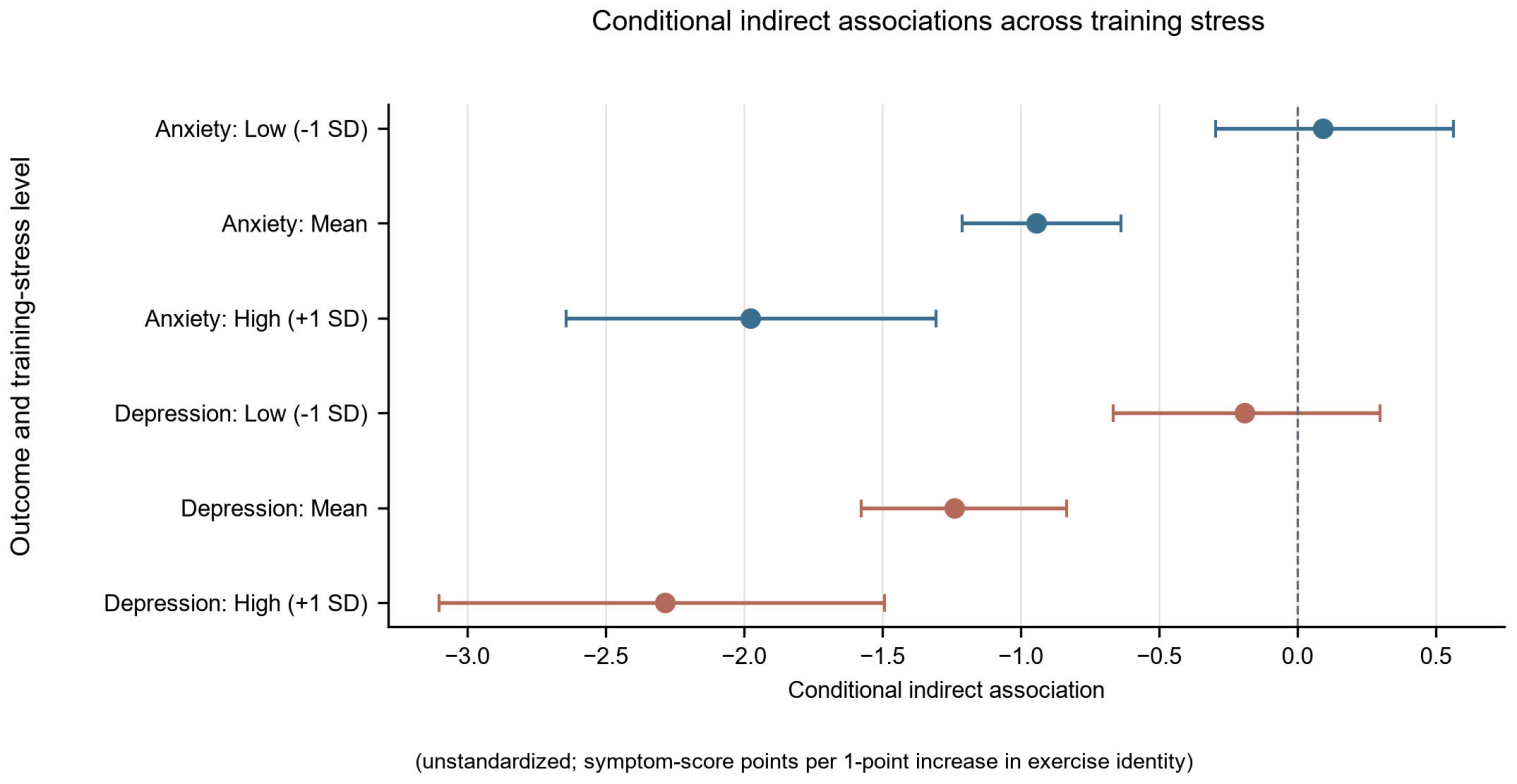
Conditional indirect associations. Point estimates and bootstrap 95% confidence intervals are shown for adjusted complete-case conditional indirect associations at low (−1 SD = 3.47), mean (4.72), and high (+1 SD = 5.98) levels of perceived training stress (*n* = 209). These are sample-derived probe values rather than validated cutoffs. Estimates are cross-sectional associations, not causal mediation effects. Unstandardized; symptom-score points per 1-point increase in exercise identity.

Sensitivity models using the full retained sample for the five core variables (*n* = 363) did not reproduce the adjusted conditional indirect pattern. In those models, exercise identity was not associated with self-compassion, *b* = 0.013, SE = 0.024, *p* = 0.579. The self-compassion by training-stress interaction was not significant for anxiety, *b* = −0.830, SE = 0.580, *p* = 0.153, or depression, *b* = 0.464, SE = 0.662, *p* = 0.484. Bootstrap indirect estimates at low, mean, and high training stress all had confidence intervals that included zero. Sensitivity coefficients are reported in Table S3, and complete-case comparisons are reported in Table S4. These models are a joint sensitivity check, not a decomposition: they changed both sample composition and covariate adjustment, so the discrepancy cannot be attributed uniquely to complete-case selection or covariate control. The adjusted complete-case evidence for H3 and H4 should therefore be treated as exploratory and specific to a higher-strain subset unless replicated in nested specifications or with appropriate missing-data methods.

## Discussion

### Principal findings

This study asked whether exercise identity among university sport participants is associated with psychological distress, and whether self-compassion and perceived training stress clarify that association. The answer is not a simple protective story. Stronger exercise identity was positively correlated with anxiety and depressive symptoms, indicating that sport-related self-definition can coexist with distress in this sample. Self-compassion was consistently associated with fewer symptoms, whereas perceived training stress was consistently associated with more symptoms at the bivariate level. These three main correlational patterns are the most stable findings. In adjusted complete-case models, exercise identity was not directly associated with anxiety but remained positively associated with depressive symptoms, and moderation and conditional indirect patterns emerged. Because those conditional patterns did not reproduce in the full core-variable sensitivity models, they are better viewed as a plausible psychological process for future testing or as a pattern specific to a higher-strain subset, not as established evidence of mediation or buffering. This interpretation fits the broader athlete mental health literature, which cautions against assuming that sport involvement alone protects young people from internalizing symptoms (Gouttebarge et al., 2019; Reardon et al., 2019; Rice et al., 2016).

### Exercise identity and psychological distress

The findings support a double-edged interpretation of exercise identity. A strong sport or exercise identity may organize routines, provide social belonging, and sustain participation, but it may also tie self-worth to training, selection, performance, and bodily readiness. This interpretation is consistent with Brewer's, Van Raalte and Linder (1993) account of athletic identity as both a source of role meaning and a possible point of vulnerability when the sport role is threatened. It also fits the exercise identity measurement tradition, which treats sport and exercise self-definition as relevant beyond formal elite-athlete samples (Anderson and Cychosz, 1994; Wilson and Muon, 2008). Recent social-identity approaches likewise emphasize that athlete identity is psychologically consequential rather than merely descriptive, particularly during transitions or periods of disrupted sport participation (Antoniak et al., 2022; Parker et al., 2021; Stevens et al., 2024). In the present sample, the positive bivariate correlations with anxiety and depression show why university sport participation should not be treated as uniformly protective. Students who define themselves strongly through sport may be especially exposed to sport-specific evaluation and loss when performance, injury, or academic conflict threatens that identity.

The adjusted models sharpen this interpretation but do not settle the psychological process. Once covariates and self-compassion were included, the direct association between exercise identity and anxiety was essentially zero, whereas the association with depression remained positive. This difference suggests that exercise identity may not have a uniform relation with all forms of internalizing symptoms. It may be more closely tied to depressive features such as loss of competence, reduced self-worth, or perceived failure when sport demands become difficult. The sensitivity results matter here: the adjusted exercise identity to self-compassion association appeared only in complete cases, who reported higher exercise identity, training stress, anxiety, and depressive symptoms than retained participants excluded for covariate missingness. The cross-sectional design and the sensitivity-model results prevent stronger claims, but the bivariate pattern is sufficient to reject the assumption that stronger exercise identity is automatically beneficial for mental health.

### Self-compassion under training stress

Self-compassion was the clearest lower-distress correlate in the model. It was negatively correlated with anxiety and depression, and the adjusted coefficients remained large after accounting for exercise identity, training stress, and sport-exposure covariates. This result is consistent with Neff's (2003) six-component formulation: greater self-kindness, common humanity, and mindfulness, together with less self-judgment, isolation, and over-identification. It also aligns with sport-specific work that connects self-compassion with athletes' responses to setbacks, injury, evaluation, and performance pressure and with lower competition anxiety (Casali et al., 2022; Cormier et al., 2025; Ferguson et al., 2014; Gano-Overway and Peterson, 2023; Mosewich et al., 2011; Wortley and Schellenberg, 2025). In a university sport context, mistakes, fatigue, and selection pressure are routine experiences. Self-compassion may mark whether those experiences are appraised as specific disappointments that can be addressed or as global judgments of personal worth.

Training stress qualified this association in the adjusted complete-case models. The significant negative interaction terms indicated that the inverse association between self-compassion and symptoms was stronger at higher perceived training stress. This pattern aligns with stress-appraisal and recovery-stress approaches that distinguish perceived demand, recovery, evaluation, and conflict from simple training volume (Kellmann and Kallus, 2001; Lemelin et al., 2022; Lu et al., 2012; Mansell, 2021). Burnout evidence points in the same direction: sustained demands, need frustration, recovery difficulty, and pressure are associated with psychological and physical costs in athletes, a pattern that fits broader self-determination accounts of how need frustration is linked with poorer wellbeing (Deci and Ryan, 2000; Glandorf et al., 2025; Li et al., 2013; Woods et al., 2025). The bivariate correlations in the present study showed that training stress was positively associated with both anxiety and depression, so the interaction should not be read as meaning that stress is beneficial. A more defensible interpretation is that self-compassion may be most visible as a lower-distress correlate when sport participation is experienced as demanding. Because the interaction was not reproduced in the core-variable sensitivity models, this moderation pattern requires replication before being treated as stable.

The conditional indirect associations extend the same point but require stronger caution. At mean and high training stress, exercise identity showed negative indirect associations with symptoms through self-compassion in the adjusted complete-case models; at low training stress, the confidence intervals crossed zero. The sensitivity models did not support the same pattern. The most defensible interpretation is therefore not that the proposed mechanism has been established, but that identity, self-compassion, and training stress may form a testable appraisal system in a higher-strain subgroup. Across the full retained sample, the data more securely support self-compassion and training stress as independent correlates of distress alongside a positive exercise identity-distress association. Because all constructs were measured concurrently, the conditional model locates a hypothesis for longitudinal testing rather than demonstrating a temporal pathway.

### Theoretical and practical implications

Theoretically, the findings can be organized within a demand-resource appraisal framework. Exercise identity increases the salience of sport outcomes: the more central sport is to the self, the more consequential performance, selection, injury, and interruption may feel. Perceived training stress represents the immediate demand context, including pressure, recovery difficulty, and academic conflict. Self-compassion represents a potential appraisal and emotion-regulation resource that may help keep a setback specific, shared, and changeable rather than global, isolating, and identity-threatening. This framework explains why a strong exercise identity can coexist with both commitment and distress, and why self-compassion may matter most when demands are high. However, the present data establish only the separate correlational roles of these constructs with confidence; the adjusted interaction and conditional indirect pattern remains provisional because it was not robust to the sensitivity specification. The contribution is therefore to identify an integrated model for stronger longitudinal testing, not to classify sport identity as inherently good or bad.

Practically, the results argue against interventions that simply encourage stronger sport identification. Campus sport programs, coaches, and student-support services may need to ask how students respond when sport becomes stressful. Screening for perceived training stress, recent injury, and internalizing symptoms may be more useful than assuming that committed participants are coping well. Self-compassion training, reflective recovery practices, and coaching climates that separate performance feedback from personal worth are plausible support targets, and they are consistent with sport self-compassion and athlete mental health intervention literatures (Ekelund et al., 2022, 2025; Mosewich et al., 2013; Myall et al., 2023; Wang et al., 2025). The practical claim must remain limited, however, because the present study cannot test intervention outcomes, screening accuracy, or within-person change.

### Limitations and future research

Several limitations constrain the manuscript. The data came from a one-wave self-report questionnaire, so common method variance, reverse-direction explanations, and unmeasured confounding remain plausible. The study-specific training-stress index showed high internal consistency, but it should not be treated as a validated instrument without further psychometric work. No *a priori* power analysis was conducted for the present models. Some covariates had missing values, reducing the complete-case sample in adjusted models from 363 to 209. Complete cases also reported higher exercise identity, training stress, anxiety, and depressive symptoms than retained participants excluded for covariate missingness, so the adjusted models should not be generalized to the full retained sample without caution. The sensitivity analysis did not reproduce the exercise identity to self-compassion association, the self-compassion by training-stress interactions, or the conditional indirect estimates when the core-variable models were estimated in the full retained sample without covariates. Because this comparison simultaneously changed sample composition and covariate specification, it cannot distinguish complete-case selection from covariate adjustment; either or both may account for the discrepancy. Nested analyses in the same complete-case sample and principled missing-data approaches would be needed to separate these explanations. Anxiety and depressive symptoms were highly correlated, indicating that the two outcomes should be read as closely related indicators of internalizing distress rather than independent endpoints. The α values for exercise identity, anxiety, depression, and training stress were very high, suggesting that future work should inspect item redundancy and dimensionality rather than treating high reliability as sufficient evidence of measurement quality.

Future research should test the model longitudinally, ideally measuring exercise identity before later training stress, self-compassion, and symptoms. A two-wave or intensive longitudinal design would allow stronger evaluation of whether self-compassion buffers later distress under high training stress. Intervention studies are also needed to determine whether self-compassion can be changed in university sport settings and whether such change reduces anxiety or depression for students experiencing heavy sport demands.

## Conclusion

Exercise identity among university sport participants was not associated with psychological distress in a simple protective pattern. Stronger exercise identity coexisted with higher anxiety and depressive symptoms at the bivariate level, self-compassion was consistently linked with lower symptoms, and perceived training stress was linked with higher symptoms. These are the most stable findings. The adjusted complete-case models suggested a stronger self-compassion-symptom association at higher perceived training stress, but this moderation and the related conditional indirect pattern were not robust in the core-variable sensitivity models and came from a higher-strain subsample. The conditional model should therefore be treated as a plausible demand-resource appraisal framework for future testing rather than an established psychological pathway. Longitudinal, missing-data-sensitive, and intervention studies are needed before temporal, directional, or practice-effectiveness claims can be made.

## Data Availability

The original contributions presented in the study are included in the article/Supplementary material, further inquiries can be directed to the corresponding authors.
