# Supplementary material for: Exercise identity and psychological distress among university sport participants: the roles of self-compassion and training stress
Source: Front Psychol. 2026 Sep 21;17:1926280. doi: 10.3389/fpsyg.2026.1926280 (PMC13634859; doi:10.3389/fpsyg.2026.1926280)
Supplement: Supplementary file 1 [file Supplementary_file_1.docx]

# Supplementary Materials

**Table S1.** *Measurement and scoring details*

| **Construct** | **Score variable** | **Items used** | **Observed score range** | **Theoretical range** | **Reliability alpha** | **Scoring note** |
| --- | --- | --- | --- | --- | --- | --- |
| Exercise identity | exercise_identity | eis_1-eis_9 | 3.67-7.00 | 1-7 | 0.981 | Mean score; higher scores indicate stronger exercise identity. |
| Self-compassion | self_compassion | scs_sf_1-scs_sf_12 | 2.42-4.67 | 1-5 | 0.787 | Mean score after reverse coding specified items; higher scores indicate greater self-compassion. |
| Perceived training stress | training_stress | ts_1-ts_5 | 3.00-7.00 | 1-7 | 0.921 | Mean score; study-specific index covering pressure, recovery difficulty, academic conflict, emotional strain, and selection/evaluation worry. |
| Anxiety symptoms | anxiety | gad_1-gad_7 | 0.00-21.00 | 0-21 | 0.975 | Sum score; higher scores indicate greater anxiety symptoms. |
| Depressive symptoms | depression | phq8_1-phq8_8 | 0.00-22.00 | 0-24 | 0.968 | Sum score; higher scores indicate greater depressive symptoms. |

**Table S2.** *Categorical response counts*

| **Variable** | **Category** | **n** | **%** |
| --- | --- | --- | --- |
| gender | Female | 190 | 52.3 |
| gender | Male | 173 | 47.7 |
| study_year | First-year undergraduate | 181 | 49.9 |
| study_year | Second-year undergraduate | 61 | 16.8 |
| study_year | Master's student | 74 | 20.4 |
| study_year | Doctoral student | 38 | 10.5 |
| study_year | Other | 9 | 2.5 |
| sport_level | Recreational participation | 215 | 59.2 |
| sport_level | University team | 70 | 19.3 |
| sport_level | Course requirement | 42 | 11.6 |
| sport_level | Other | 36 | 9.9 |
| sport_type | Team sport | 131 | 36.1 |
| sport_type | Individual sport | 120 | 33.1 |
| sport_type | Mixed team/individual sport | 49 | 13.5 |
| sport_type | Fitness or conditioning | 39 | 10.7 |
| sport_type | Other | 24 | 6.6 |
| competition_status | Yes | 103 | 28.4 |
| competition_status | No | 260 | 71.6 |

*Note.* Categories are translated from the questionnaire response options. Percentages use the retained analytic sample as the denominator.

**Table S3.** *Sensitivity comparison of unadjusted core-variable and adjusted complete-case models*

| **Specification** | **Model** | **Term** | **b** | **SE** | **t** | **p** |
| --- | --- | --- | --- | --- | --- | --- |
| Unadjusted model | Self-compassion | Exercise identity | 0.013 | 0.024 | 0.555 | 0.579 |
| Unadjusted model | Anxiety | Exercise identity | 1.111 | 0.193 | 5.747 | < .001 |
| Unadjusted model | Anxiety | Self-compassion | -5.047 | 0.41 | -12.299 | < .001 |
| Unadjusted model | Anxiety | Training stress | 2.509 | 0.21 | 11.936 | < .001 |
| Unadjusted model | Anxiety | Self-compassion x training stress | -0.83 | 0.58 | -1.431 | 0.153 |
| Unadjusted model | Depression | Exercise identity | 1.799 | 0.221 | 8.154 | < .001 |
| Unadjusted model | Depression | Self-compassion | -4.856 | 0.468 | -10.368 | < .001 |
| Unadjusted model | Depression | Training stress | 1.957 | 0.24 | 8.159 | < .001 |
| Unadjusted model | Depression | Self-compassion x training stress | 0.464 | 0.662 | 0.701 | 0.484 |
| Adjusted complete-case model | Self-compassion | Exercise identity | 0.095 | 0.02 | 4.724 | < .001 |
| Adjusted complete-case model | Anxiety | Exercise identity | 0.054 | 0.311 | 0.175 | 0.861 |
| Adjusted complete-case model | Anxiety | Self-compassion | -9.97 | 0.777 | -12.828 | < .001 |
| Adjusted complete-case model | Anxiety | Training stress | 0.577 | 0.319 | 1.81 | 0.072 |
| Adjusted complete-case model | Anxiety | Self-compassion x training stress | -8.734 | 1.282 | -6.812 | < .001 |
| Adjusted complete-case model | Depression | Exercise identity | 0.769 | 0.303 | 2.533 | 0.012 |
| Adjusted complete-case model | Depression | Self-compassion | -13.098 | 0.758 | -17.268 | < .001 |
| Adjusted complete-case model | Depression | Training stress | -0.922 | 0.311 | -2.965 | 0.003 |
| Adjusted complete-case model | Depression | Self-compassion x training stress | -8.84 | 1.251 | -7.065 | < .001 |

**Table S4.** *Complete-case comparison on core study variables*

| **Variable** | **Complete-case n** | **Complete-case M** | **Complete-case SD** | **Excluded n** | **Excluded M** | **Excluded SD** | **Mean difference** | **t** | **p** |
| --- | --- | --- | --- | --- | --- | --- | --- | --- | --- |
| Exercise identity | 209 | 5.934 | 1.094 | 154 | 5.113 | 1.092 | 0.821 | 7.072 | < .001 |
| Self-compassion | 209 | 3.412 | 0.499 | 154 | 3.373 | 0.552 | 0.039 | 0.701 | 0.484 |
| Perceived training stress | 209 | 4.724 | 1.252 | 154 | 4.116 | 0.666 | 0.609 | 5.974 | < .001 |
| Anxiety symptoms | 209 | 9.675 | 6.006 | 154 | 5.701 | 5.276 | 3.973 | 6.684 | < .001 |
| Depressive symptoms | 209 | 9.297 | 6.031 | 154 | 5.039 | 5.639 | 4.258 | 6.902 | < .001 |

*Note.* Complete cases are participants retained in the adjusted primary models. Excluded cases are retained analytic-sample participants who lacked at least one adjusted-model covariate. Mean differences are complete-case minus excluded-case means; p values are from Welch two-sample tests.

**Figure S1. Sample flow**

*Note.* Flow diagram from submitted responses to the retained analytic sample.

**Figure S2. Correlation heatmap**

*Note.* Heatmap of Pearson correlations among the five core study variables.

**Figure S3. Model-implied self-compassion by training-stress interaction**

*Note.* Predicted anxiety and depressive symptoms are plotted from the 5th to 75th percentile of observed self-compassion at low, mean, and high levels of perceived training stress, with covariates held at sample means.
